# Haploidentical hematopoietic stem cell transplantation as individual treatment option in pediatric patients with very high-risk sarcomas

**DOI:** 10.3389/fonc.2023.1064190

**Published:** 2023-02-21

**Authors:** Thomas Eichholz, Michaela Döring, Stefano Giardino, Bernd Gruhn, Christian Seitz, Tim Flaadt, Wolfgang Schwinger, Martin Ebinger, Ursula Holzer, Markus Mezger, Heiko-Manuel Teltschik, Monika Sparber-Sauer, Ewa Koscielniak, Michael Abele, Rupert Handgretinger, Peter Lang

**Affiliations:** ^1^ University Children’s Hospital, Eberhard Karls University, Tuebingen, Germany; ^2^ Hematopoietic Stem Cell Transplantation Unit, Department of Hematology and Oncology, IRCCS Istituto Giannina Gaslini, Genoa, Italy; ^3^ Department of Pediatrics, Jena University Hospital, Jena, Germany; ^4^ Department of Pediatrics and Adolescent Medicine, Medical University of Graz, Graz, Austria; ^5^ Klinikum der Landeshauptstadt Stuttgart gKAöR, Olgahospital, Stuttgart Cancer Center, Zentrum für Kinder-, Jugend- und Frauenmedizin, Pädiatrie 5 (Pädiatrische Onkologie, Hämatologie, Immunologie), Stuttgart, Germany; ^6^ University Tübingen, Medical Faculty, Tübingen, Germany

**Keywords:** haploidentical hematopoietic stem cell transplantation, pediatric sarcoma, Ewing sarcoma, soft tissue sarcoma, rhabdomyosarcoma

## Abstract

**Background:**

Prognosis of children with primary disseminated or metastatic relapsed sarcomas remains dismal despite intensification of conventional therapies including high-dose chemotherapy. Since haploidentical hematopoietic stem cell transplantation (haplo-HSCT) is effective in the treatment of hematological malignancies by mediating a graft versus leukemia effect, we evaluated this approach in pediatric sarcomas as well.

**Methods:**

Patients with bone Ewing sarcoma or soft tissue sarcoma who received haplo-HSCT as part of clinical trials using CD3+ or TCRα/β+ and CD19+ depletion respectively were evaluated regarding feasibility of treatment and survival.

**Results:**

We identified 15 patients with primary disseminated disease and 14 with metastatic relapse who were transplanted from a haploidentical donor to improve prognosis. Three-year event-free survival (EFS) was 18,1% and predominantly determined by disease relapse. Survival depended on response to pre-transplant therapy (3y-EFS of patients in complete or very good partial response: 36,4%). However, no patient with metastatic relapse could be rescued.

**Conclusion:**

Haplo-HSCT for consolidation after conventional therapy seems to be of interest for some, but not for the majority of patients with high-risk pediatric sarcomas. Evaluation of its future use as basis for subsequent humoral or cellular immunotherapies is necessary.

## Introduction

Ewing sarcoma (ES) together with soft tissue and other extraosseous sarcomas accounted for 8% of malignancies in children <18 years between 2009 and 2018 in Germany (1). Despite advanced multimodal therapies, the prognosis of these entities remains poor in the case of primary disseminated or relapsed disease.

The five-years overall survival (OS) of children with disseminated rhabdomyosarcoma (RMS) is estimated between 20-30% (2, 3), reducing to less than 20% in relapsed disease (4). Nevertheless, this group of patients is not uniform and in presence of recognized risk factors such as the age older than 10 years with bone or bone marrow metastasis, the 5-year EFS falls lower than 2% (5). Alveolar histology, age of over 10 or below 1 year, bone or bone marrow metastasis, and the site of primary tumor have been also found to affect prognosis (2, 6). These conditions enter into the Oberlin-score, which defines different prognostic subgroups. An Oberlin score >2 is associated with a 3-year EFS of 14% (7).

Similarly, the prognosis of patients with disseminated or early relapsed ES is poor, with a 5-year OS reported lower than 15% (8). The age of over 14 years, bone metastasis and multiple metastases, initial tumor volume >200ml (9) and relapse within two years (10) in ES are the recognized unfavorable prognostic parameters.

For other non-rhabdomyosarcoma soft tissue sarcomas (NRSTS), like synovial sarcoma, unsatisfactory survival rates have been reported in Stage IV (11) or relapsed disease (12) as well, making them eligible for experimental therapies (13).

Despite their chemosensitivity, dose escalation with autologous stem cell rescue did not clearly result in better survival in RMS or ES. While one study found a better survival of high-dose chemotherapy in high-risk localized ES (14), other studies did not verify an improvement in survival either in RMS (6), nor ES (15, 16), and new alternative treatment options are needed in very high-risk situations for relapsed disease with very poor prognosis.

Since a graft-versus-tumor (GvT) effect could be shown in some other solid tumors (17), also in pediatric sarcomas allogeneic hematopoietic stem cell transplantation (allo-HSCT) has been seen as a potential alternative treatment option (18). Different case reports have suggested a GvT effect even in pediatric RMS and ES (5, 19–23), supporting this strategy. The development of efficient graft manipulation techniques and a reduced intensity conditioning regimen (RIC) lead to overcoming the main risks for transplant-related mortality even in haploidentical settings (24, 25), by potentially facilitating a higher GvT effect (24, 26) mediated by cell subsets preserved in the T-depleted grafts and avoiding the limiting post-transplant immunosuppressive treatments. An antitumoral effect of natural killer (NK) – cells against pediatric solid tumor cells in haploidentical setting was shown *in vitro* (27).

Here we present a retrospective analysis of children affected by RMS, ES or NRSTS who underwent an HSCT from a haploidentical donor after T-cell negative selection in 3 centers (Tübingen, Jena, and Graz).

## Materials and methods

Data from children with a diagnosis of RMS, ES or NRSTS who underwent haplo-HSCT from 2005 to 2019 have been retrospectively collected. Patients were enrolled in clinical trials about haplo-HSCT with T-cell depletion in pediatric diseases in that period with CD3+/CD19+ (Tübingen, Jena, Graz) [ClinicalTrials.gov NCT01919866] or TCRα/β+ and CD19+ negative selection (Tübingen). Patients with RMS, ES or NRSTS and primary bone metastasis or bone marrow involvement, with relapsed metastatic or primary refractory disease to standard treatment were eligible for haplo-HSCT. Patients have been in part already published in another context (28, 29). Data analysis was done as of August 2022.

The patients have been considered eligible for this analysis in presence of a diagnosis ES, RMS or other STS at high risk because of stage IV disease at diagnosis or relapse. Data regarding diagnosis, disease status before transplant, pre-HSCT treatments, conditioning regimen, donor features, graft manipulation, engraftment, transplant-related toxicity/morbidity, acute (aGvHD) and chronic graft-versus-host disease (cGvHD), disease status after HSCT, and survival have been retrospectively collected if not already present in the databases of the above-mentioned clinical trials.

We evaluated overall survival (OS) and event-free survival (EFS) as well as the incidence of transplant-related mortality (TRM), grade II-IV aGvHD, extensive cGvHD and relapse.

*Data analysis and definitions:* Stage IV disease has been defined as the presence of distant metastasis at diagnosis. Complete response was defined as the disappearance of all visible disease on imaging, partial response (PR) as a reduction of at least 30% (ES) or 33% (RMS/NRSTS) of tumor volume and progressive disease (PD) as an increase in tumor volume of more than 20% (ES) or 33% (RMS/NRSTS) or the appearance of new lesions according to the respective treatment protocols. Nonresponse was defined as a tumor response between PR and PD. Very good partial response (VGPR) was defined as a reduction of ≥90 of tumor volume or the persistence of unclear residuals upon imaging. Engraftment was defined as the first of three consecutive days with an absolute leukocyte count (ALC) of more than 1000/µl. GvHD was diagnosed and graded according to Glucksberg criteria (30). A failure to achieve ALC >1000/µl until day 28 posttransplant defined primary graft failure (PGF), whereas a decline of ALC <1000/µl after initial engraftment and not caused by infection, drug toxicity or relapse defined secondary graft failure.

The probability of survival from the date of transplantation to death/last follow-up was defined as overall survival (OS). In one patient who was rescued with a syngeneic stem cell graft after rejection of her haploidentical graft, TRM, cumulative incidence of relapse, GvHD or virus reactivation, EFS and OS were censored at the timepoint of subsequent transplantation. Every death, not caused by relapse or progress of the underlying disease was accounted as transplant-related. Events were defined as relapse, death in remission or secondary malignancy, whichever occurred first. The Kaplan-Meier method was used to estimate the probability of survival, using GraphPad Prism version 7 for Windows software (GraphPad Software, Inc., San Diego, California USA, www.graphpad.com). Calculation of cumulative incidence of time to relapse, time to ADV or CMV virus reactivation, time to GvHD and time to treatment related death accounting for the concurrent risk “death”, “relapse” and “non-treatment related death”, respectively were done by SAS using Cox regression.

## Results

We identified 29 patients eligible for this analysis, with diagnoses of ES (n=14), alveolar rhabdomyosarcoma (aRMS) (n=12), botryoid rhabdomyosarcoma (n=1), undifferentiated sarcoma (n=1) or synovial sarcoma (n=1) who received haplo-HSCT in centers involved (Tübingen n=23, Jena n=5, Graz n=1).

All patients were considered to be at very high risk due to stage IV disease at diagnosis (n=23) and/or relapse (n=14) and eligible for haplo-HSCT. In all but one patient with aRMS, the Oberlin-score was ≥2. Eight patients with ES presented with multiple bone metastasis at diagnosis and 8 relapsed within 2 years after initial diagnosis, before they were considered for haplo-HSCT. Patient characteristics are summarized in **Table 1**.

**Table 1:** Patient Characteristics.

	Total	Ewing Sarcoma	Rhabdomyosarcoma	Non-rhabdomyosarcoma soft tissue sarcoma
No of patients (%)	29	14 (48,3%)	Alveolar RMS	Undifferentiated sarcoma
			12 (41,4%)	1 (3,4%)
			Botryoid RMS	Synovial sarcoma
			1 (3,4%)	1 (3,4%)
Age at diagnosis	12,2	12,9	7,5	10,96
years median (range)	(1 – 23,8)	(7,9 – 17,7)	(1 – 23,8)	(10,2 – 11,8)
Diagnosis (%)				
• Primary stage IV	15 (51,7%)	5	9	1
• Metastatic relapse	14 (48,3%)	9	4	1
Previous therapies				
• Radiotherapy	19 (65,5%)	10	7	2
• Autologous/syngeneic HSCT	9 (31%)	7	2	0
• other	Samarium 3	Samarium 1	Samarium 2	
	mTOR Inhibitor 3	mTOR Inhibitor 2	mTOR Inhibitor 1	
	TKI 1	TKI 1		
	Anti-IGF2-mAb 1	Anti-IGF2-mAb 1		
	Avastin 1		Avastin 1	
	HIPEC 1		HIPEC 1	
Remission status prior to HSCT (%)				
• CR 1	6 (20,7%)	3	2	1
• CR ≥2	2 (6,9%)	0	1	1
• PR/VGPR 1	9 (31%)	2	7	0
• PR/VGPR ≥2	6 (20,7%)	4	2	0
• NR	6 (20,7%)	5	1	0
HSCT, depletion technique (%)				
• CD3+/CD19+	20 (69%)	11	7	2
• TCRα/β+ / CD19+	9 (31%)	3	6	0

Initial therapy complied with the ongoing clinical trials and recommendations at the time of diagnosis. Patients with ES were treated either according Euro-E.W.I.N.G.99 or EWING-2008 study respectively. All but two patients with RMS, as well as the patients with unspecified sarcoma and synovial sarcoma were treated according to the studies and recommendations of the CWS study group, including CWS-96, CWS IV-2002, CWS DOK IV 2004, CWS-2002P and CWS guidance. Of the remaining two patients with rhabdomyosarcoma, one received initial treatment in the United States according to the recommendations of the children´s oncology group (COG). The other one was treated according to EWING-2008, since the primary diagnosis was ES but changed to aRMS after relapse. The majority of patients (15; 51,7%) responded to initial therapy and entered transplantation in first complete remission (CR1, n=6), very good partial response (VGPR1, n=3) or partial response (PR1, n=6). Another 8 patients (27,6%) responded after previous relapse or progression (CR≥2 n=2; PR≥2 n=6). Six patients were transplanted without response (NR) (20,7%).

*Conditioning regimen*: Apart from treosulfan- and busulfan-based conditioning regimens in two and one patient, a toxicity reduced regimen was deployed in most transplants, using fludarabin (160 mg/m² body surface area) in 24 or clofarabin (200mg/m²/d) in 2 cases, in combination with melphalan (140 mg/m²) and thiotepa (10 mg/kg). Two patients with previous haplo-HSCT and graft failure received additional TLI (4 Gy respectively 7 Gy) to reduce the risk of another graft failure.

Serotherapy was part of the conditioning regimen in all patients, comprising OKT3 (given day -9 until day +17) until 2010 (n=17) and ATG (given day -11 until day -9) since OKT3 was no longer available in 2011 (n=12).

*Stem cell source and graft manipulation*: Mobilized peripheral blood stem cells (PBSC) using granulocyte colony-stimulating factor (Granocyte^®^, Lenograstim, Chugai Pharma; Neupogen^®^, Filgrastim, Amgen) were the stem cell sources harvested from haploidentical family donors. Parents served as stem cell donors in 26 transplants, mismatched adult siblings (n=2) and aunt (n=1) in the remaining. The degree of HLA-mismatch (MM) ranged from 2 mismatches in one case, 3 MMs in 4 patients, 4 MMs in 8 patients, and 5 MM in 16 HSCTs. Grafts were processed with CD3+ and CD19+ depletion (n=20) or TCRα/β+ and CD19+ depletion (n=9) respectively, using the automated CliniMACS^®^ system (Miltenyi Biotec, Bergisch Gladbach, Germany) as described earlier (31). The graft composition is specified in **Table 2**.

**Table 2:** Graft Composition.

	CD34+ *10^6^/kg	CD3+ § *10³/kg	TCRα/β+ ≠ *10³/kg	TCRγ/δ+ ≠ *10^6^/kg	CD19+ *10³/kg	CD56+ *10^6^/kg
minimum	2,57	9,5	8,4	1,5	8,508	23,42
median	11,2	41,33	12,48	9,13	36,12	56,78
maximum	26,13	100,02	35,81	20,85	561,031	298,26
number of patients	29	20	9	9	23	23

In case of reconditioning 1^st^ graft considered. **§** only CD3+/CD19+ depleted grafts considered. ≠ only TCRα/β+ / CD19+ depleted grafts considered.

*Engraftment:* Primary engraftment occurred in 27 patients after a median of 10 days (range 7-15), while 2 patients (CD3+/CD19+ n=1, TCRα/β+ / CD19+ depletion n=1) suffered from primary graft failure (6,9%). Both patients could be rescued, using an immunoablative reconditioning regimen, comprising either fludarabin, thiotepa with or without cyclophosphamide, ATG and TLI as described earlier (32), followed by a second haploidentical graft with CD3+/CD19+ or TCRα/β+ / CD19+-depletion respectively from a different donor. Three patients showed a secondary rejection (secondary graft failure 10,3%). Two patients received an autologous backup 29 and 38 days after HSCT respectively. Both were found to be eligible for another haplo-HSCT and were transplanted with a second CD3+/CD19+ depleted graft 7 months after the first one and further follow-up was counted from the second haplo-HSCT. One of these patients suffered another relapse by that time. The third patient with secondary graft failure was rescued with a graft from a syngeneic sibling 85 days after haplo-HSCT since she received a syngeneic HSCT already before haplo-HSCT.

*Immune reconstitution:* Data were available for 23 patients. In patients, who received a subsequent haploidentical graft following graft failure, the immune phenotype was counted from the day the second graft was infused.

**Figure 1:**
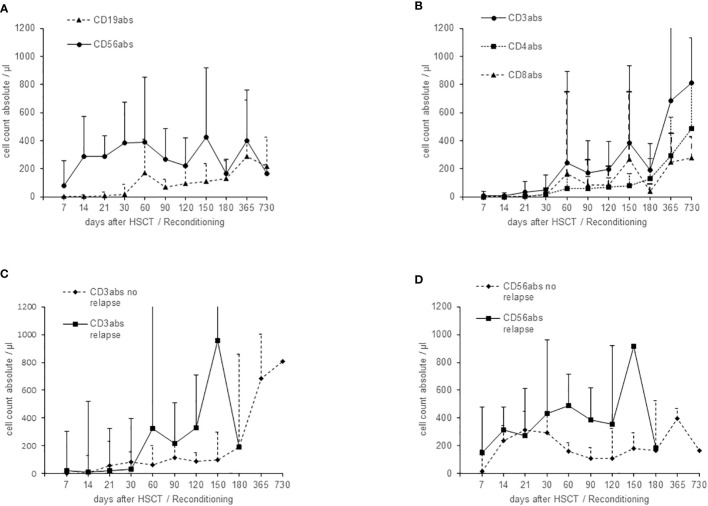
Immune reconstitution after haploidentical HSCT (mean and standard deviation), calculated from total lymphocyte cell count and flow cytometry results. **(A)** Reconstitution of CD56+ NK and CD19+ B cells. **(B)** Reconstitution of CD3+ T cells (CD4+ and CD8+). **(C)** Comparison of the recovery of CD3+ T cells in patients with or without relapse. **(D)** Comparison of the recovery of CD56+ NK cells in patients with or without relapse.

Recovery of CD56+ natural killer (NK) cells was fast, reaching counts of >100cells/µl mean already at day 14. Reconstitution of CD3+ lymphocytes started at day +30, although cell counts of CD3+ T-lymphocytes at day 90 were below 200/µl (172/µl, range 3 – 728) and CD3+CD8+ below 100/µl (87/µl, range 0 – 601) (**Figures 1A, B**). Reconstitution of CD3+, or CD56+ lymphocytes was not faster in patients without relapse (**Figures 1C, D**).

**Figure 2:**
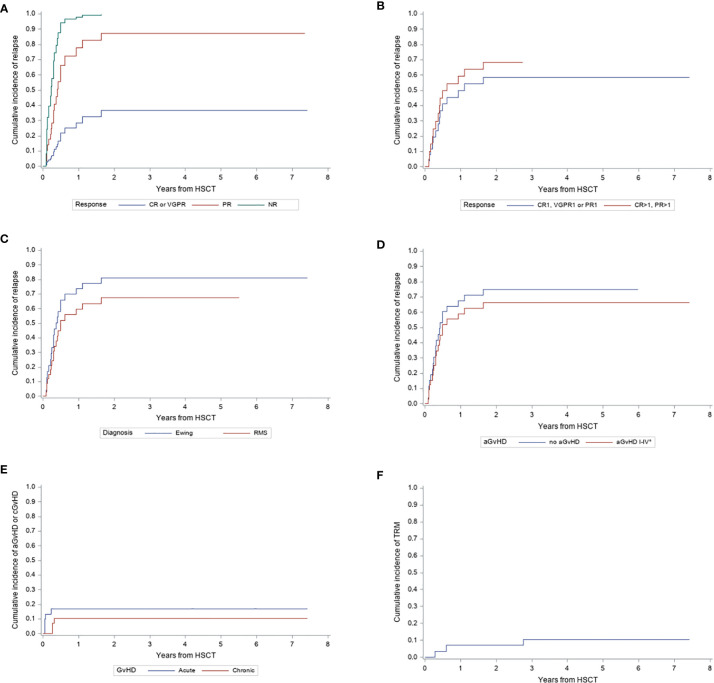
Cumulative Incidence (CI) of relapse, GvHD and transplant related mortality from haploidentical HSCT. **(A)** CI of relapse in patients who reach CR or VGPR after pre-transplant therapy compared to patients with either PR or NR. **(B)** CI of relapse in patients achieving first CR, VGPR or PR pre-transplant compared to patients in CR or PR who were transplanted after relapse. **(C)** CI of relapse in patients with Ewing Sarcoma or Rhabdomyosarcoma. **(D)** CI of relapse according to occurrence of aGvHD °I-IV or no aGvHD. **(E)** CI of aGvHD °II-IV and cGvHD. **(F)** CI of transplant related mortality.

*Graft-versus-host disease:* Mycophenolate mofetil (MMF) was the only GvHD prophylaxis in all patients (standard dosage of 1200 mg/m² in two single dosage). Acute GvHD was overall diagnosed in 17 patients, two of them after receiving post-transplant DLI due to mixed chimerism. Only 5 patients (3-years CI 16,8%) developed clinically relevant GvHD grade II or higher (II° n=1, III° n=3, IV° n=1). Three patients (3-years CI 10,4%) developed cGvHD (limited disease n=1, extended disease n=2) (**Figure 2E**).

*Relapse:* Relapse occurred in 20 patients (ES n=12, alveolar RMS n=7, botryoid RMS n=1) at median 112 days (36 days – 1,6 years) post haplo-HSCT, resulting in a cumulative incidence (CI) of 69% after 3 years. Patients, who responded well on pre-transplant treatment (CR or VGPR) suffered significantly less from relapse (3-years CI of relapse 36,8%) than patients with PR (CI 87,0%, p=0,01) or NR (CI 99,5%, p=0,0003) (**Figure 2A**). While the CI of relapse was lower in patients transplanted in first CR, VGPR or PR, the difference to patients with CR or PR after previous relapse was not statistically significant (58,6% vs. 68,3%; p=0,62) (**Figure 2B**). Similarly, we didn’t find significant difference in CI of relapse by diagnosis between ES and RMS (ES 80,9% vs. RMS 67,7%; p=0,39) (**Figure 2C**). None of the patients with NRSTS relapsed. However, this group comprises only 2 patients of whom one died from GvHD 33 months after HSCT. There was no significant difference in CI of relapse in patients who developed aGvHD of any grade compared to those who did not experience aGvHD (no aGvHD 74,8% vs. aGvHD °I-IV 66,4%, p=0,59) (**Figure 2D**).

*Viral reactivation*: CMV viremia was detected in 4 patients (3 years CI 13,9%) and ADV viremia in 6 patients (3 years CI 20,6%). ADV infection caused 1 death. There was no patient experiencing post-transplant lymphoproliferative disease.

*Transplant-related mortality*: Three deaths were attributed to transplantation procedure (ADV infection, GvHD with sepsis, intracranial bleeding), resulting in a TRM of 10,5% after 3 years (**Figure 2F**).

**Figure 3:**
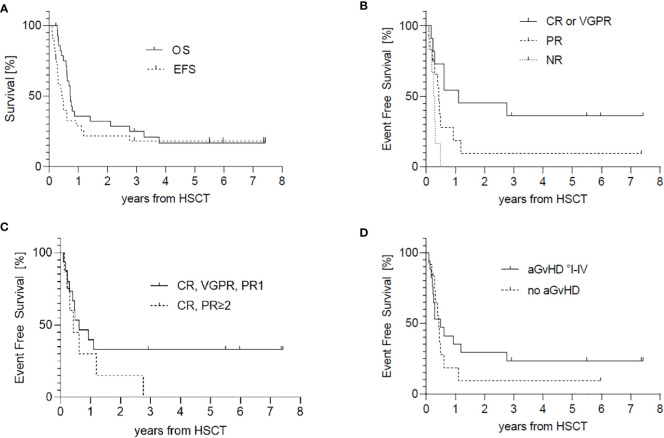
Survival, counted from haploidentical HSCT. **(A)** Probability of Overall Survival (OS) and Event Free Survival of the entire cohort. **(B)** EFS according to pre-transplant remission status CR/VGPR vs. PR or NR. **(C)** EFS of patients achieving first PR or better compared to patients in CR/PR, who were transplanted after relapse. **(D)** EFS in accordance with the occurrence of aGvHD.

*Survival:* The 5-year-OS probability was 16,1% (**Figure 3A**). Details of the five surviving patients are summarized in **Table 3**.

**Table 3:** Details of surviving patients.

	Patient I	Patient II	Patient III	Patient IV	Patient V
Diagnosis	Ewing sarcoma	aRMS	aRMS	Undifferentiated sarcoma	Ewing sarcoma
Stage at diagnosis	Stage IV Multiple bone metastases	Stage IV Multiple bone metastases	Stage IV Multiple bone & pelvic & nodal metastases	Stage IV Nodal & pulmonal metastases	Stage IV Multiple bone & nodal metastases
Pre-Transplant Remission status	CR1	VGPR1	VGPR1	CR1	PR1
Conditioning regimen	Treosulfan, Fludarabin, Thiotepa, OKT3	Melphalan, Fludarabin, Thiotepa, ATG	Melphalan, Fludarabin, Thiotepa, ATG, TLI	Melphalan, Fludarabin, Thiotepa, OKT3	Melphalan, Fludarabin, Thiotepa, ATG
Form of graft manipulation	CD3+/CD19+ depletion	CD3+/CD19+ depletion	CD3+/CD19+ depletion	CD3+/CD19+ depletion	TCRα/β+/CD19+ depletion
Donor No of Mismatch	Father 4/10	Father 5/10	Mother 5/10	Mother 5/10	Father 4/10
Chimerism at d100 either CD3, PBMC	Complete	Complete	Complete	Complete	Complete
aGvHD, max. grade	Grade 1	Grade 1	Grade 1	No aGvHD	Grade 3
cGvHD	No cGvHD	No cGvHD	No cGvHD	No cGvHD	No cGvHD
Last follow up in years after HSCT	7,4	5,5	2,9	5,97	7,4

The EFS probability was 18,1% after 3 years (**Figure 3A**) and dependent only on pre-transplant remission status. Patients with at least VGPR experienced lesser events than patients with only partial or worse response (EFS of patients in CR or VGPR (36,4%) vs. PR (9,4%; p=0,08) or NR (0%; p=0,01) (**Figure 3B**). Patients with first partial or better response fare better than patients who already experienced relapse pre-transplant. The difference did not reach statistical significance. None of the patients with previous relapse survived regardless of response to pre-transplant treatment. (EFS of patients in first CR, VGPR or PR 33,3% vs. subsequent CR or PR 0%; p=0,2) (**Figure 3C**). The occurrence of aGvHD did not significantly influence EFS (EFS without aGvHD 9,3% vs. aGvHD I-IV° 23,5%; p=0,45) (**Figure 3D**). Whether cGvHD developed or not did not affect EFS, whereby patients might have experienced relapse before cGvHD could have developed. The major events were relapse in 19 patients (82%) followed by transplant-related deaths (n=3). In one patient a secondary neoplasm occurred (vulvar intraepithelial neoplasm) 14 months after haplo-HSCT, before relapse of the primary Ewing sarcoma.

## Discussion

Haplo-HSCT with selective T-cell depletion has been used in hematological malignancies with impressive results (33) and it is also in part reported in some patients with solid tumors (5, 27). These approaches could have the advantages of providing high doses of cell subsets that mediate the anti-tumor effect through HLA independent pathways without carrying a graft-versus-host effect, such as NK cells that prevail in CD3+-negative selected graft as in the early stages after engraftment. In the more recent TCRα/β+-negative selection, the TCRγ/δ+ lymphocytes, preserved in the graft, further enhance the GvT effect as well as carry out a fundamental antiviral activity (34). Moreover, since in this context prolonged immunosuppression after transplant is not required, the donor-derived T cells activity, whose reconstitution occurs later, is not hindered.

However, while T-cell depleted haploidentical HSCT was safe, in this study it was applicable for patients with response but not effective for patients with advanced pediatric sarcomas not in response after initial therapy.

These results have to be interpreted in the context of the extremely high-risk profile of our cohort. Almost half of our patients (48,3%) experienced one or subsequent metastatic relapses pre-transplant, which occurred early within 2 years after primary diagnosis in 10 patients, especially with ES. More than 20% of patients underwent transplantation in non-response, and another 12 patients (41%) responded just partially before HSCT and have been transplanted with significant residual tumor mass, with consequent increased risk of later relapse or progression. Only 38% of patients achieved CR or VGPR prior to HSCT. The influence of pre-transplant remission status on survival has been reported earlier in patients with Ewing sarcoma (29). Indeed, in our cohort, patients who underwent haplo-HSCT in presence of better remission status (CR or VGPR) showed a significantly lower cumulative incidence of relapse (CIR) and a better EFS than those with PR (CIR 36,8% vs 87,0%, EFS 36,4% vs 9,4%) or persistent disease (CIR 99,5%, EFS 0%) (**Figures 2A**, **3B**). This points up the fundamental role of pre-transplant approaches in pediatric sarcoma, as reported in other malignancies (35). A total of 5 patients survived without signs of disease. All of them received a haplo-HSCT in CR1, VGPR1 or PR 1, whereas patients transplanted after relapse could not be rescued. This raises the question if a haplo-HSCT could be a treatment option as consolidation therapy following standard treatment protocols for primary disease, especially in patients with complete or very good partial response or if low dose maintenance chemotherapy will be equal or more effective. Carli et al. reported an EFS of 23% in patients reaching CR after conventional therapy with a prolonged course of chemotherapy in RMS (6). Otherwise, Klingebiel et al. reported an OS of 52% in patients with metastatic soft tissue sarcomas receiving oral maintenance therapy (36). Another matched pair analysis showed no benefit of allogeneic HSCT over non transplanted controls (37) and Merker et al. (38) found no benefit of haplo-HSCT compared to reported results of oral maintenance therapy. However, randomized studies addressing this issue are missing. To achieve statistical relevant patient numbers, a European or even international study would be necessary, although, the feasibility of a randomized study is questionable because of the poor prognosis in this cohort of patients.

Relapse occurred early, within a median of 112 days post-HSCT and thus before sufficient reconstitution of T-cells, probably making too short the timing interval for their contribution to the GvT-effect (**Figure 1B**). Choosing a more intense conditioning regimen might have prevented early relapse (39) and thus given time for a T-cell mediated GvT effect to fully develop. However, as most of our patients did not respond completely to intense pre-transplant chemotherapy regimen, we regard this effect limited. Furthermore, therapy related toxicity is likely to cause a higher TRM rate in this heavily pretreated patient group and outweigh any benefit from a more intense conditioning.

Despite NK-cells being recovered within two weeks after HSCT, confirming previous observations in T-depleted haplo-HSCT for both malignant (40, 41) and non-malignant diseases (34, 42), and the immunosuppression only being short-term, GvT-effect apparently was not sufficient for the majority of patients in our cohort of pediatric sarcomas. GvHD prophylaxis using MMF was given over a short period of 30d median. Since NK-cell recovery was fast, MMF seems not to have impaired NK-cell proliferation but it cannot be excluded that MMF might have impaired NK-cell function (43) and thus possible GvT- effects in our setting. Beside this, a retrospective analysis by Thiel et al. also could not show a clear GvT-effect in most patients with Ewing sarcoma neither in HLA-mismatched nor in HLA-matched HSCT (29).

Apart from NK-cells, a GvT-effect is often attributed to donor T-cells. Childs et al. reported a regression of metastatic solid tumors after establishment of a complete T-cell chimerism and withdrawal of immunosuppression or infusion of donor lymphocytes (17). Since T-cell reconstitution takes time following T-cell depletion, full GvT-effect is delayed as well. Previous reports on haplo-HSCT in pediatric sarcomas based mainly on T-cell depletion (5, 24, 29, 38). An alternative to T-cell depletion in the setting of haplo-HSCT could be the use of T-cell-repleted grafts. However, the use of unmanipulated grafts in haplo-HSCT requires an intense GvHD-prophylaxes (post-transplantation cyclophosphamide or Beijing protocol). Haplo-HSCT using T-cell replete grafts constitutes an interesting approach, however, it has to be determined if the use T-cell-repleted grafts results in a stronger GvT-effect. So far none of the different transplantation protocols was superior over the others regarding OS or relapse (44).

This work has limitations and strengths. The main limitations consist of the retrospective analysis and the small number and heterogeneity of diagnosis of patients included. Furthermore, the inclusion of patients with no response to initial therapy can be questioned. However, this study offers the opportunity to address important questions about haplo-SCT in a subgroup of pediatric solid tumors with poor prognosis.

Based on the above-mentioned results, haploidentical HSCT seems not to considerably improve outcome in most patients with high-risk Ewing sarcoma and RMS, particularly without good response on initial treatment. This observation corresponds with our experience in relapsed neuroblastoma (45). Nevertheless, dose-escalation of conventional therapy including high-dose chemotherapy reached its limits without substantial improvement in survival (15). Therefore, targeted therapies and immunotherapies remain an area of special interest in pediatric sarcomas, as for other cancers. There are hints, that IL2 given posttransplant can augment donor derived NK cell activity in a rather unspecific way (26, 46). On the other hand, another study about prophylactic IL2 administration found an increased risk of relapse (47). Thus, more specific immunotherapies are needed. Based on the experience in leukemias and neuroblastomas, treatment with appropriate monoclonal antibodies (mAb) or antibody-cytokine fusion proteins post-transplant, chimeric antigen receptor (CAR)-T-cell therapy and more recently CAR-NK-cells from the stem cell donor might be potential therapeutic approaches in the future (48). The identification of a tumor-antigen with features of high expression on tumor cells and low expression on healthy tissues remains the main challenge for an effective and safe clinical translation of such approaches. Different possible targets for CAR T-cells or mAbs are currently under investigation in sarcomas, including HER2 (49, 50), B7-H3 (51), ErbB2 (52), GD2 (53, 54), or VEGFR2 (55). The possibility to apply an antibody, CAR-T or CAR-NK approach based on the donor derived immune system in a haploidentical context could represent a fascinating option for the future.

The high safety profile of haploidentical HSCT with CD3+/CD19+ (56) or TCRαβ+/CD19+ depletion (28), carrying on low GvHD-rate and TRM, makes it feasible even in heavily pretreated patients, representing an ideal basis for potential subsequent immunotherapies.

## Data availability statement

The original contributions presented in the study are included in the article/supplementary materials, further inquiries can be directed to the corresponding author/s.

## Ethics statement

Ethical review and approval was not required for the study on human participants in accordance with the local legislation and institutional requirements. Written informed consent from the participants’ legal guardian/next of kin was not required to participate in this study in accordance with the national legislation and the institutional requirements.

## Author contributions

PL and TE conceived the idea of this work. PL, RH and EK designed the clinical trials in which the reported patients participated. PL, RH, MA, H-MT, MM, UH, ME, WS, TF, CS, BG, MD and TE contributed to the acquisition and analysis of data. TE, MD and SG drafted the manuscript with the help of the other authors. PL, MS-S, ME, EK, and TF critically revised the manuscript. All authors contributed to the article and approved the submitted version.
